# Melanoma of the penis with scintigraphically-guided sentinel node biopsy

**DOI:** 10.4103/0970-1591.70587

**Published:** 2010

**Authors:** William H. Tu, Denise Johnson, Harcharan Gill

**Affiliations:** Department of Urology, USA; 1 Surgery, Stanford University School of Medicine, Stanford, USA

**Keywords:** Melanoma, sentinel lymph node, penis

## Abstract

Melanoma of the penis is an uncommon cancer. We present the case of a 73-year-old male with penile melanoma and non palpable lymph nodes. Lymphoscintigraphy was applied to locate the sentinel lymph nodes for dissection. His lymph nodes were negative for melanoma and he has been disease-free for 1 year with careful surveillance.

## INTRODUCTION

Penile cancer is uncommon in the United States but has a higher incidence in Africa and Asia. The most common type is squamous cell carcinoma, which is associated with being uncircumcised, chronic inflammation, phimosis, and the human papilloma virus.[1] Melanoma of the penis is rare but more than 100 cases have been reported. Nevertheless, management of patients with nonpalpable lymph nodes remains controversial.[2,3] Bilateral superficial inguinal lymph node dissections have been the gold standard but the risks include lymphedema, phlebitis, and infection.[2] Sentinel lymph node biopsy has been proposed as a less morbid alternative. We describe a case of penile melanoma and the utility of scintigraphy to lateralize sentinel lymph nodes for dissection.

## CASE REPORT

A 73-year-old uncircumcised male with a history of melanoma *in situ*, squamous cell carcinoma, and basal cell carcinoma of the head, neck, and extremities presented to his local doctor for a red bump on his penis noted during self-examination. He denied any tenderness, ulceration, discharge, or bleeding from the lesion. His medical history was also significant for Merckel cell carcinoma on the left side of his face metastatic to lymph nodes that were treated with a left superficial parotidectomy, modified radical neck dissection, and radiation therapy. He did not receive chemotherapy for the Merckel cell carcinoma and has had no evidence of recurrent disease. The penile lesion was non pigmented, flat, hyperaemic, and measured 4×5 mm. It was located on the dorsum of the shaft of his penis close to the corona of the glans. He was treated with several courses of antibiotics over 4 months without a significant change in the lesion. An excisional biopsy was performed under local anaesthesia and revealed a nodular malignant melanoma, Breslow depth 2.5 mm, Clark Level IV, positive for ulceration, with a negative deep margin but a positive lateral margin. He was referred to us for further evaluation and treatment.

On physical examination, he was healthy with well healed surgical scars of the head, neck, and extremities. No inguinal adenopathy was noted on palpation. The external genitalia showed an uncircumcised penis, and on retraction of the foreskin there was a partially healed surgical incision on the dorsum of the shaft of the penis close to the corona of the glans. A computed tomography-positron emission tomography (CT-PET) scan showed no evidence of metastasis. Based upon these findings, he underwent a wide local excision, essentially a radical circumcision, and sentinel lymph node biopsy for further staging.

**Figure 1:**
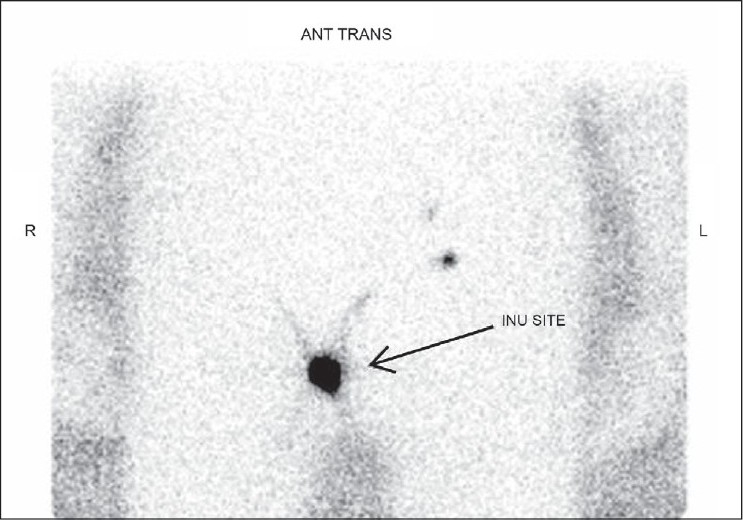
Lymphoscintigraphy lateralizing the sentinel nodes to the left groin

One hour preoperatively, the patient received an intradermal injection of technetium-99m filtered sulfur colloid around the prior biopsy site on the foreskin for sentinel lymph node scintigraphy. The flow images identified a sentinel lymph node in the left groin only [Figure 1]. Wide excision of the bed of the previous biopsy, circumcision, and excision of the scintigraphically positive nodes was done under anaesthesia. Intraoperatively, a Neoprobe was used to identify the sentinel node and confirm removal of all radioactive tissue.

**Figure 2:**
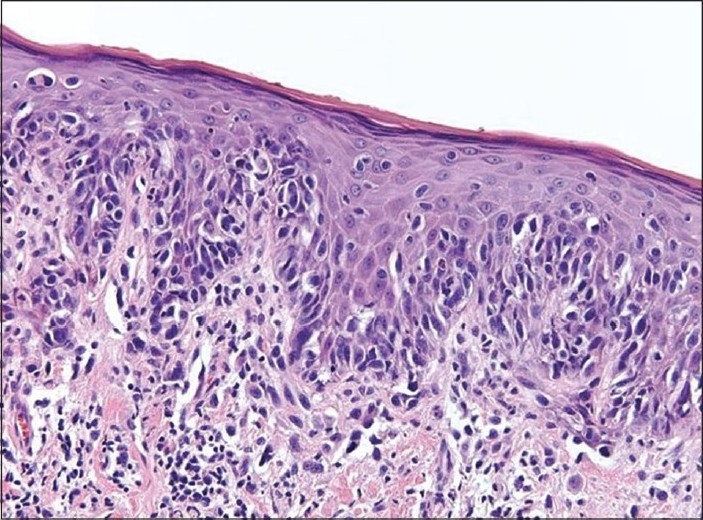
Hematoxylin and eosin staining showing melanoma *in situ* (H and E, ×20)

The pathology specimen showed no residual invasive melanoma but only melanoma *in situ* [Figure 2] that was immunoreactive to Pan-melanoma, Melan-A, vimentin and S100. The four lymph nodes were negative for malignancy. Given the melanoma *in situ*, the patient was treated with imiquimod topical 5% cream three times per week for 3 weeks by his dermatologist. He had no evidence of local or regional recurrence at the 3 year follow-up visit.

## DISCUSSION

Melanoma is the fifth most common cancer in men, but the incidence of primary penile involvement is only about 1%.[4,5] The cutaneous cancer arises from melanocytes and can be aggressive with a poor prognosis especially in the case of deep invasion. Early diagnosis is important for successful treatment with surgical resection.

The most common penile tumor is squamous cell carcinoma, which has a higher incidence in men who are uncircumcised. Squamous cell carcinoma is differentiated from melanoma on histopathology of the biopsy and immunohistochemical staining for common melanoma markers. The rarity of penile melanoma makes the management open to debate. The primary treatment of the lesion is surgical, which may be wide local excision, a partial penectomy, or a total penectomy. Melanoma localized to the foreskin may be treated with circumcision alone. All patients with palpable lymph nodes that do not resolve after antibiotic treatment should undergo inguinal lymph node dissection.[4] However, management of patients with non palpable lymph nodes is controversial. In other cutaneous melanomas, the standard treatment is sentinel node identification and biopsy for both staging and therapy. In this patient, we used a scintigraphic technique to identify the sentinel nodes and to spare him the morbidity of bilateral inguinal lymph node dissection. Due to the location of the tumor, it was not possible to lateralize without this study. The prognosis of melanoma of the penis is similar to other cutaneous melanoma and depends on the size and depth of the tumor, but sentinel node information is helpful for staging and can be predictive of recurrence.[4,5]

Scintigraphy is done easily with minimal morbidity and provides easy localization of non palpable regional sentinel nodes. However, there is a risk of false negative findings and thus careful follow-up is necessary. In our case, the patient has had negative surveillance imaging and negative physical examinations every 3-6 months for 3 years and we were able to spare the patient bilateral inguinal node dissection.

## References

[1] Bleeker MC, Heidman DA, Snijders PJ, Horenblas S, Dillner J, Meijer CJ Penile cancer: epidemiology, pathogenesis, and prevention. World J Urol Jul.

[2] Hankins CL, Weston P (2006). Re: Melanoma of the penis, scrotum and male urethra: a 40-year single institution experience. J Urol.

[3] Spiess PE, Izawa JI, Bassett R, Kedar D, Busby JE, Wong F (2007). Preoperative lymphoscintigraphy and dynamic sentinel lymph node biopsy for staging penile cancer: results with pathological correlation. J Urol.

[4] Sanchez-Ortiz R, Huang SF, Tamboli P, Prieto VG, Hester G, Pettaway CA (2005). Melanoma of the penis, scrotum and male urethra: a 40-year single institution experience. J Urol.

[5] van Geel AN, den Bakker MA, Kirkels W, Horenblas S, Kroon BB, de Wilt JH (2007). Prognosis of primary mucosal penile melanoma: a series of 19 Dutch patients and 47 patients from the literature. Urology.

